# Predictive accuracy of combined genetic and environmental risk scores

**DOI:** 10.1002/gepi.22092

**Published:** 2017-11-26

**Authors:** Frank Dudbridge, Nora Pashayan, Jian Yang

**Affiliations:** ^1^ Department of Health Sciences University of Leicester Leicester United Kingdom; ^2^ Department of Non‐Communicable Disease Epidemiology London School of Hygiene and Tropical Medicine London United Kingdom; ^3^ Department of Public Health and Primary Care University of Cambridge Cambridge United Kingdom; ^4^ MRC Biostatistics Unit University of Cambridge Cambridge United Kingdom; ^5^ Department of Applied Health Research University College London London United Kingdom; ^6^ Institute for Molecular Bioscience University of Queensland Brisbane Queensland Australia; ^7^ Queensland Brain Institute University of Queensland Brisbane Queensland Australia

**Keywords:** polygenic score, reclassification, risk prediction, risk score, ROC curve

## Abstract

The substantial heritability of most complex diseases suggests that genetic data could provide useful risk prediction. To date the performance of genetic risk scores has fallen short of the potential implied by heritability, but this can be explained by insufficient sample sizes for estimating highly polygenic models. When risk predictors already exist based on environment or lifestyle, two key questions are to what extent can they be improved by adding genetic information, and what is the ultimate potential of combined genetic and environmental risk scores? Here, we extend previous work on the predictive accuracy of polygenic scores to allow for an environmental score that may be correlated with the polygenic score, for example when the environmental factors mediate the genetic risk. We derive common measures of predictive accuracy and improvement as functions of the training sample size, chip heritabilities of disease and environmental score, and genetic correlation between disease and environmental risk factors. We consider simple addition of the two scores and a weighted sum that accounts for their correlation. Using examples from studies of cardiovascular disease and breast cancer, we show that improvements in discrimination are generally small but reasonable degrees of reclassification could be obtained with current sample sizes. Correlation between genetic and environmental scores has only minor effects on numerical results in realistic scenarios. In the longer term, as the accuracy of polygenic scores improves they will come to dominate the predictive accuracy compared to environmental scores.

## 1 INTRODUCTION

Predicting the individual risk of disease is one of the major goals of epidemiology and, because most common diseases have a heritable component, there has long been interest in using genotype data to inform prediction of disease onset, prognosis, or treatment response. Indeed for Mendelian disorders genetic prediction has established clinical applications in counselling, prophylactic intervention, and embryonic screening. For the common, complex disorders however, progress has to date been slow (Abraham & Inouye, 2015; Chatterjee, Shi, & Garcia‐Closas, 2016; Jostins & Barrett, 2011). Despite the success of genome‐wide association studies (GWAS) in identifying numerous risk variants for many disorders, these variants typically explain a small proportion of the variation in risk; a number of studies have examined the predictive accuracy of GWAS “hits” and generally found limited utility for risk prediction (Eriksson et al., 2015; Talmud et al., 2015; Weissfeld et al., 2015), although there have been some successes (Maas et al., 2016; Patel et al., 2016). The reasons are that the associated markers individually have small effects on risk, and the markers discovered to date are a small fraction of the total complement of risk variants. The realization that most, perhaps all, complex traits are polygenic, that is, determined by thousands of genetic variants with small effects, has motivated a shift toward thinking about the genetic basis of disease as a single entity (Dudbridge, 2016). Under this paradigm, the whole genome should be regarded as a risk predictor, but attempts in this direction have so far also yielded modest results (Evans, Visscher, & Wray, 2009; Locke et al., 2015; Maier et al., 2015).

However, given the substantial heritability of many common disorders, it remains true that we should improve risk prediction if we could measure the heritable component accurately (Pharoah et al., 2002; Wray, Goddard, & Visscher, 2007; Wray, Yang, Goddard, & Visscher, 2010). Indeed, a crude measure of polygenic risk, namely family history, is already widely used as a risk predictor (Valdez, Yoon, Qureshi, Green, & Khoury, 2010). An important insight is that the currently disappointing performance of polygenic prediction can be explained by insufficient sample sizes available for estimating genetic effects (Chatterjee et al., 2013; Dudbridge, 2013). Essentially, sampling variation accumulates across thousands of variants so that the polygenic predictor has high measurement error even if standard errors are low on each individual variant. However, international consortium efforts and national biobank projects are now approaching the sizes at which accurate genetic predictors could be derived, so the performance of genetic prediction may well improve toward useful levels in the next few years.

Nevertheless, the heritability imposes a limit on the accuracy of genetic prediction, even if we knew all the variants affecting risk and their precise effect sizes (Clayton, 2009). For many disorders, this limit falls short of levels usually regarded as clinically useful, and the only way we could improve predictive accuracy would be by adding further nongenetic factors into the risk score. Indeed for some conditions, for example, cardiovascular disease (CVD) and breast cancer, epidemiological risk scores are already available and in clinical use. The question then is whether inclusion of genetic information could improve the predictive accuracy to a useful degree. As with studies of genetic prediction alone, efforts to improve existing predictors by adding in genetic data have met with only moderate success (Morris et al., 2016; Talmud et al., 2015; Wacholder et al., 2010).

Theoretical treatments of genetic prediction have mainly considered prediction from genotypes alone, but not the common scenario in which both genetic and nongenetic predictors are combined. An exception is work of Garcia‐Closas, Gunsoy, and Chatterjee (2014), who argue that addition of genetic data to questionnaire‐based risk factors could provide useful levels of risk stratification for breast cancer. Here, we extend previous work on the predictive accuracy of polygenic risk scores (Dudbridge, 2013) to include a nonpolygenic component. For convenience, we term all nonpolygenic risk factors as “environmental,” although such factors could include major genes, behavioral and other factors such as age that arguably do not fit that description. A key concern is that many environmental risk factors are themselves heritable, and may therefore mediate some or all of the genetic risk. Indeed, comparable resources have been directed toward GWAS of heritable risk factors such as lipid fractions (Willer et al., 2013) and body mass index (BMI) (Locke et al., 2015) as have been toward clinical outcomes. On the other hand, for environments that are difficult to measure well (e.g., alcohol consumption), it is conceivable that their heritable component may act as a more accurate predictor of the outcome than the measured environment itself (Verhulst, Neale, & Kendler, 2015).

Here, we show how under a quantitative genetics model, commonly used measures of predictive accuracy and improvement can be expressed in terms of the genetic variance of an outcome of interest (its “chip heritability”), genetic variance of an environmental risk score, variance in the outcome explained by the environmental score, and genetic correlation between the outcome and the environmental score. These are quantities that are readily estimated by existing methods (Bulik‐Sullivan et al., 2015; Lee, Yang, Goddard, Visscher, & Wray, 2012), allowing examples to be drawn from recent literature to illustrate the prospects for prediction from combined genetic and environmental factors. As in previous work (Dudbridge, 2013), we allow for the estimation of a polygenic risk score from finite training data, but in contrast to other work (Garcia‐Closas et al., 2014; So & Sham, 2010), we explicitly consider the genetic correlation between environment and outcome and its implications for the computation of combined risk scores.

## 2 MATERIALS AND METHODS

### 2.1 Quantitative Model

Consider two linear models for an outcome *Y*, the first in terms of a scalar environmental factor *X*:

E(Y)=X

the second in terms of *m* genetic markers:

$$E\left(Y\right)=\beta^{\prime }G=\sum_{i=1}^{m}\beta_{i}G_{i},$$

where β is an *m*‐vector of coefficients $\beta_{i}$. Assume that the effect of the environmental factor *X* on *Y* is known precisely, so that without loss of generality *X* may be assumed to have a unit effect on *Y*. Denote by $R_{XY}^{2}$ the proportion of variance in *Y* explained by *X*. Assuming also that *Y* is standardized, then $\mathrm{var}\left(X\right)=R_{XY}^{2}$.

Assume that genotypes G are independent (i.e., in linkage equilibrium) and standardized. Thus, for single nucleotide polymorphisms (SNPs) in Hardy–Weinberg equilibrium, the usual additive coding gives $G_{i}=\left(A_{i}-2f_{i}\right)/{\left(2f_{i}\left(1-f_{i}\right)\right)}^{1/2}$, where $A_{i}$ is the number of minor alleles and $f_{i}$ is the minor allele frequency at SNP *i*. The genetic effects $\beta_{i}$ are regarded as fixed across samples, but random over i=1,…,m with $E(\beta_{i})=0$, $\mathrm{var}\left(\beta_{i}\right)=m^{-1}\sigma_{G}^{2}$. Then the genetic variance of *Y* is $\sigma_{G}^{2}$, and because *Y* is standardized the chip heritability of markers G, denoted by $R_{GY}^{2}$, is equal to $\sigma_{G}^{2}$.

The separate marginal genetic and environmental models for *Y* reflect common practice in fitting such models. Many environmental risk factors have been extensively studied prior to the genomics era, and accurate estimates of their effects have been obtained from large epidemiological studies without adjustment for genetic factors. Here it is assumed that the environmental effect size is known with negligible standard error so that we may ignore sampling error in the effect of *X*.

Genetic effect sizes are often estimated by large consortia in which it may be impractical to adjust for any but the simplest risk factors (e.g., age and sex) across contributing studies. However, estimation of the marginal genetic effects is a key determinant of predictive accuracy. Let the genetic effects be estimated from a training sample of size *n* with the polygenic risk score then defined by:

$$\hat{S}={\hat{\beta }}^{\prime }G.$$

For ordinary least squares estimates of β, Dudbridge (Dudbridge, 2013) showed that:

(1) $$\begin{array}{c} \mathrm{cov}\left(\hat{S},Y\right)=2R_{GY}^{2}\left[\Phi \left(r_{0}\right)-\Phi \left(r_{1}\right)+r_{1}\varphi \left(r_{1}\right)-r_{0}\varphi \left(r_{0}\right)\right], \end{array}$$

(2) $$\begin{array}{ccc} \mathrm{var}(\hat{S}) & = & 2m\pi_{0}n^{-1}\left[\Phi \left(q_{0}\right)-\Phi \left(q_{1}\right)+q_{1}\varphi \left(q_{1}\right)-q_{0}\varphi \left(q_{0}\right)\right]\\ \; & \; & +2m\left(1-\pi_{0}\right)\left({\left(1-\pi_{0}\right)}^{-1}m^{-1}R_{GY}^{2}+n^{-1}\right)\\ & & \;\left[\Phi \left(r_{0}\right)-\Phi \left(r_{1}\right)+r_{1}\varphi \left(r_{1}\right)-r_{0}\varphi \left(r_{0}\right)\right], \end{array}$$

where π_0_ is a proportion of markers assumed to have no effect ($\beta_{i}=0$), with the rest assumed to have effects following a normal distribution; $q_{0}=\Phi^{-1}\left(1-\frac{1}{2}p_{0}\right)$, $q_{1}=\Phi^{-1}\left(1-\frac{1}{2}p_{1}\right)$, where $p_{0},p_{1}$ are lower and upper two‐tailed *P*‐values to select markers into the polygenic score; and $r_{0}=q_{0}{\left(n{\left(1-\pi_{0}\right)}^{-1}m^{-1}R_{GY}^{2}+1\right)}^{-\frac{1}{2}}$, $r_{1}=q_{1}{\left(n{\left(1-\pi_{0}\right)}^{-1}m^{-1}R_{GY}^{2}+1\right)}^{-\frac{1}{2}}$.

Now consider a polygenic component to the environmental factor *X*. Denote the chip heritability of *X* by $R_{GX}^{2}$ and the genetic correlation between *X* and *Y* by ρ. That is, ρ is the correlation between the $\beta_{i}$ and the corresponding genetic effects on *X*: thus it is a property of the markers G and should properly be called a chip correlation. Then the chip covariance between *X* and *Y* is:

$$\rho \sqrt{m^{-2}R_{GY}^{2}R_{XY}^{2}R_{GX}^{2}}$$

and so

$$\mathrm{cov}\left(S,X\right)=m\rho \sqrt{m^{-2}R_{GY}^{2}R_{XY}^{2}R_{GX}^{2}}=\rho \sqrt{R_{GY}^{2}R_{XY}^{2}R_{GX}^{2}}.$$

The covariance of the estimated polygenic score $\hat{S}$ with the environmental score *X* also follows from the results of Dudbridge:

(3) cov (S^,X)=2 cov (S,X)Φ(r0)−Φ(r1)+r1φ(r1)−r0φ(r0)=2ρRGY2RXY2RGX2Φ(r0)−Φ(r1)+r1φ(r1)−r0φ(r0).

Now consider a combined score formed as the weighted sum of polygenic and environmental scores:

(4) $${\hat{S}}_{comb}=w_{1}\hat{S}+w_{2}X.$$

The coefficient of determination for the combined score is:

(5) RS^combY2= cov (S^comb,Y)2 var (S^comb),

where

(6) cov (S^comb,Y)=w1 cov (S^,Y)+w2RXY2,

(7) var (S^comb)=w12 var (S^)+w22RXY2+2w1w2 cov (S^,X).

A simple choice of weights just adds the two scores together, $w_{1}=w_{2}=1$. Although this seems naive when there is genetic correlation between *X* and *Y*, it is a very commonly used approach. A better choice is to take the ordinary least squares solution to the linear model:

$$E\left(Y\right)={\hat{S}}_{comb},$$

which is

w1w2= var (S^) cov (S^,X) cov (S^,X) var (X)−1 cov (S^,Y) cov (X,Y)= var (S^) cov (S^,X) cov (S^,X)RXY2−1 cov (S^,Y)RXY2.

To summarize thus far, the coefficient of determination for the combined score is expressed in terms of the genetic model parameters listed in Table 1 and the derived quantities $\mathrm{cov}(\hat{S},Y)$, $\mathrm{var}(\hat{S})$, and $\mathrm{cov}(\hat{S},X)$ (Equations (1), (2), and (3)). For continuous *Y*, this coefficient of determination is the *R* ^2^ between predicted and observed traits, a common measure of predictive accuracy.

**Table 1:** Parameters and Notation of Polygenic Model

Design Parameters	Interpretation
* n*	Training sample size
* m*	Total number of independent markers in genotyping panel
* p*_0_, *p*_1_	Lower and upper *P* values to select markers into polygenic score
**Genetic model parameters**	
* * $R_{GY}^{2}$	Variance in *Y* explained by genotypes **G**; chip heritability of *Y*
* * $R_{XY}^{2}$	Variance in *Y* explained by environment *X*
* * $R_{GX}^{2}$	Variance in *X* explained by genotypes **G**; chip heritability of *X*
* *ρ	Genetic (chip) correlation between *X* and *Y*
* *π_0_	Proportion of markers with no effect on *Y*

### 2.2 Binary Outcomes

For binary outcomes, the liability threshold (or probit) model closely approximates the linear model for the small effects expected on polygenic traits, and allows several measures of predictive accuracy to be expressed analytically (So & Sham, 2010). Let *Y* now be an unobserved liability, distributed as standard normal, with the binary outcome *D *= 1 when *Y* exceeds a fixed threshold τ, 0 otherwise. Denote by *K* the prevalence of the trait and by *P* the case/control sampling ratio in the training data, defined to equal *K* in a prospective study. The variance of the observed *D* in the training data is P(1−P) and effects on *Y* are transformed to effects on *D* by the factor $c=\varphi \left(\tau \right)\frac{P(1-P)}{K(1-K)}$ (Lee, Wray, Goddard, & Visscher, 2011). Let $\hat{S}$ now be the polygenic score with effects estimated on the observed *D* and transformed back to the liability scale by $c^{-1}$. Then analogous to Equations (1), (2), and (3),

$$\mathrm{cov}\left(\hat{S},Y\right)=2R_{GY}^{2}\left[\Phi \left(r_{0}\right)-\Phi \left(r_{1}\right)+r_{1}\varphi \left(r_{1}\right)-r_{0}\varphi \left(r_{0}\right)\right],$$

$$\begin{array}{ccc} \mathrm{var}(\hat{S}) & = & c^{-2}2m\pi_{0}P\left(1-P\right)n^{-1}\\ & & \left[\Phi \left(q_{0}\right)-\Phi \left(q_{1}\right)+q_{1}\varphi \left(q_{1}\right)-q_{0}\varphi \left(q_{0}\right)\right]\\ & & +2mc^{-2}\left(1-\pi_{0}\right)({\left(1-\pi_{0}\right)}^{-1}m^{-1}c^{2}R_{GY}^{2}\\ & & +P\left(1-P\right)n^{-1})\left[\Phi \left(r_{0}\right)-\Phi \left(r_{1}\right)+r_{1}\varphi \left(r_{1}\right)\right.\\ & & \left.-\;r_{0}\varphi \left(r_{0}\right)\right], \end{array}$$

where now

$$r_{0}=q_{0}{\left(nP^{-1}{\left(1-P\right)}^{-1}{\left(1-\pi_{0}\right)}^{-1}m^{-1}c^{2}R_{GY}^{2}+1\right)}^{-\frac{1}{2}},$$

$$r_{1}=q_{1}{\left(nP^{-1}{\left(1-P\right)}^{-1}{\left(1-\pi_{0}\right)}^{-1}m^{-1}c^{2}R_{GY}^{2}+1\right)}^{-\frac{1}{2}}.$$

The combined score ${\hat{S}}_{comb}$ is calculated as before from Equation (4) and its coefficient of determination $R_{{\hat{S}}_{comb}Y}^{2}$ from Equations (5), (6), and (7).

### 2.3 Area under ROC Curve

The accuracy of predicting a binary outcome is often assessed by the area under the receiver operator characteristic curve (AUC), which measures the discrimination concordance between risk scores and outcomes. That is, AUC is the probability that a subject with the trait has a higher risk score than a subject without the trait. The central limit theorem implies that the polygenic score $\hat{S}$ is normally distributed. Assuming that the environmental score *X* also is normally distributed:

(8) $$AUC=\Phi \left(\frac{E\left({\hat{S}}_{comb}|D=1\right)-E\left({\hat{S}}_{comb}|D=0\right)}{\sqrt{\mathrm{var}\left({\hat{S}}_{comb}|D=1\right)+\mathrm{var}\left({\hat{S}}_{comb}|D=0\right)}}\right),$$

where

$$E\left({\hat{S}}_{comb}|D=1\right)=\frac{\varphi (\tau )}{K}R_{{\hat{S}}_{comb}Y}^{2},$$

$$\begin{array}{ccc} & & \mathrm{var}({\hat{S}}_{comb}|D=1)\\ & & \;=R_{{\hat{S}}_{comb}Y}^{2}\left[1-\frac{\varphi (\tau )}{K}R_{{\hat{S}}_{comb}Y}^{2}\left(\frac{\varphi (\tau )}{K}-\tau \right)\right], \end{array}$$

$$E\left({\hat{S}}_{comb}|D=0\right)=\frac{-\varphi (\tau )}{1-K}R_{{\hat{S}}_{comb}Y}^{2},$$

$$\begin{array}{ccc} & & \mathrm{var}({\hat{S}}_{comb}|D=0)\\ & & \;=R_{{\hat{S}}_{comb}Y}^{2}\left[1-\frac{\varphi (\tau )}{1-K}R_{{\hat{S}}_{comb}Y}^{2}\left(\frac{\varphi (\tau )}{K}+\tau \right)\right]. \end{array}$$

Full derivations of these equalities have been provided previously (Dudbridge, 2013; So & Sham, 2010; Wray et al., 2010).

### 2.4 Net Reclassification Index

The AUC has some limitations that have been well documented (Cook, 2007), notably that it reflects only the ranking of subjects, but not their absolute risk levels. Furthermore, substantial increases in AUC may be hard to achieve even with the addition of informative markers. This has led to the proposal of new measures of incremental predictive accuracy, of which the net reclassification improvement (NRI) has proved popular (Pencina, D'Agostino, D'Agostino, & Vasan, 2008). Given discrete risk categories defined by absolute risk thresholds, the NRI compares the classification of subjects under an initial risk predictor to their reclassification under an augmented predictor including an additional marker. Specifically, the NRI is calculated in cases as the proportion correctly reclassified to a higher risk category minus the proportion incorrectly reclassified to a lower category. Similarly, the NRI is calculated in controls as the proportion correctly reclassified to a lower category minus the proportion incorrectly reclassified to a higher category. The case and control NRI could be combined into an overall NRI (Kerr et al., 2014; Pencina, D'Agostino, & Steyerberg, 2011).

Currently, there is substantial interest in whether a polygenic risk score adds worthwhile information to an existing environmental score. Under the liability threshold model, the NRI can be calculated from tail probabilities of the joint distribution of liability, environmental, and combined scores (So & Sham, 2010). Here the calculation is adjusted to allow for chip correlation between environment *X* and liability *Y*.

Consider two categories of risk defined by a threshold *a*. (This calculation can be extended if desired to a greater number of categories.) Under the liability threshold model, the risk for environmental score *X* is:

(9) $$1-\Phi \left(\frac{\tau -X}{\sqrt{1-R_{XY}^{2}}}\right)$$

and so the risk equals the threshold *a* when

$$X=a_{X}=\tau -\sqrt{1-R_{XY}^{2}}\Phi^{-1}\left(1-a\right).$$

The combined score, though constructed as an estimate of liability, may have variance differing from 1 depending on the choice of the weights $w_{1},w_{2}$. To obtain absolute risks from the combined score, it is therefore calibrated to the liability by rescaling by its regression coefficient on *Y*:

$$\gamma =\frac{\mathrm{cov}({\hat{S}}_{comb},Y)}{\mathrm{var}({\hat{S}}_{comb})}$$

calculated from Equations (6) and (7), so that

var (γS^comb)= cov (S^comb,Y)2 var (S^comb)=RS^combY2.

(The least squares solutions of $w_{1},w_{2}$ do give γ=1.) The risk equals the threshold *a* when

$$\gamma {\hat{S}}_{comb}=a_{S}=\tau -\sqrt{1-R_{{\hat{S}}_{comb}Y}^{2}}\Phi^{-1}\left(1-a\right).$$

Again assuming that both polygenic and environmental scores are normally distributed, the joint distribution of liability, *X*, and the calibrated combined score is trivariate normal with mean zero and variance‐covariance matrix:

1RXY2RS^combY2RXY2RXY2 cov (γS^comb,X)RS^combY2 cov (γS^comb,X)RS^combY2,

where

$$\mathrm{cov}\left(\gamma {\hat{S}}_{comb},X\right)=\gamma \left(\mathrm{cov}\left(\hat{S},X\right)+R_{XY}^{2}\right)$$

is calculated using Equation (3).

The case NRI is:

$$\begin{array}{ccc} & & \mathrm{Pr}(\gamma {\hat{S}}_{comb}>a_{S},X\leq a_{X}|D=1)\\ & & \;-\mathrm{Pr}(\gamma {\hat{S}}_{comb}\leq a_{S},X>a_{X}|D=1), \end{array}$$

where

$$\begin{array}{ccc} & & \mathrm{Pr}(\gamma {\hat{S}}_{comb}>a_{S},X\leq a_{X}|D=1)\\ & & \;=\int_{\tau }^{\infty }\int_{-\infty }^{a_{X}}\int_{a_{S}}^{\infty }\varphi_{3}\left(l,x,s\right)dsdxdl/K, \end{array}$$

$$\begin{array}{ccc} & & \mathrm{Pr}(\gamma {\hat{S}}_{comb}\leq a_{S},X>a_{X}|D=1)\\ & & \;=\int_{\tau }^{\infty }\int_{a_{X}}^{\infty }\int_{-\infty }^{a_{S}}\varphi_{3}\left(l,x,s\right)dsdxdl/K, \end{array}$$

φ_3_ being the trivariate normal density function just defined.

Similarly the control NRI is:

$$\begin{array}{ccc} & & \mathrm{Pr}(\gamma {\hat{S}}_{comb}\leq a_{S},X>a_{X}|D=0)\\ & & \;-\mathrm{Pr}(\gamma {\hat{S}}_{comb}>a_{S},X\leq a_{X}|D=0), \end{array}$$

where

$$\begin{array}{ccc} & & \mathrm{Pr}(\gamma {\hat{S}}_{comb}\leq a_{S},X>a_{X}|D=0)\\ & & \;=\int_{-\infty }^{\tau }\int_{a_{X}}^{\infty }\int_{-\infty }^{a_{S}}\varphi_{3}\left(l,x,s\right)dsdxdl/\left(1-K\right), \end{array}$$

$$\begin{array}{ccc} & & \mathrm{Pr}(\gamma {\hat{S}}_{comb}>a_{S},X\leq a_{X}|D=0)\\ & & \;=\int_{-\infty }^{\tau }\int_{-\infty }^{a_{X}}\int_{a_{S}}^{\infty }\varphi_{3}\left(l,x,s\right)dsdxdl/\left(1-K\right). \end{array}$$

### 2.5 Continuous NRI and IDI

The discrete categories used in the NRI may be a limitation when such thresholds have not been fixed in practice or when comparing predictors between studies with different characteristics. Two alternatives that do not rely on discrete categories are the continuous NRI (Pencina et al., 2011) and the integrated discrimination improvement (IDI) (Pencina et al., 2008).

In cases, the continuous NRI is the proportion whose risk score increases under the augmented predictor minus the proportion whose score decreases. Similarly in the controls it is the proportion whose risk score decreases minus that whose score increases. Therefore, in cases the continuous NRI is:

$$\begin{array}{ccc} & & \mathrm{Pr}\left(\gamma {\hat{S}}_{comb}>X|D=1\right)-\mathrm{Pr}\left(\gamma {\hat{S}}_{comb}\leq X|D=1\right)\\ & & \;=2\mathrm{Pr}(\gamma {\hat{S}}_{comb}>X|D=1)-1, \end{array}$$

where

$$\mathrm{Pr}\left(\gamma {\hat{S}}_{comb}>X|D=1\right)=\int_{\tau }^{\infty }\int_{0}^{\infty }\varphi_{2}\left(l,x\right)dxdl/K$$

with φ_2_ the bivariate normal density of liability and $\gamma {\hat{S}}_{comb}-X$. This distribution has mean 0 and variance‐covariance matrix:

1RS^combY2−RXY2RS^combY2−RXY2RS^combY2+RXY2−2 cov (γS^comb,X).

Similarly in controls, the continuous NRI is:

$$2\int_{-\infty }^{\tau }\int_{-\infty }^{0}\varphi_{2}\left(l,x\right)dxdl/\left(1-K\right)-1.$$

The IDI is the mean change in risk among cases minus the mean change in controls:

$$\begin{array}{c} E_{{\hat{S}}_{comb}}\left[1-\Phi \left(\frac{\tau -\gamma {\hat{S}}_{comb}}{\sqrt{1-R_{{\hat{S}}_{comb}Y}^{2}}}\right)|D=1\right]-E_{X}\left[1-\Phi \left(\frac{\tau -X}{\sqrt{1-R_{XY}^{2}}}\right)|D=1\right]\\ \;-E_{{\hat{S}}_{comb}}\left[1-\Phi \left(\frac{\tau -\gamma {\hat{S}}_{comb}}{\sqrt{1-R_{{\hat{S}}_{comb}Y}^{2}}}\right)|D\;=0\right]+E_{X}\left[1-\Phi \left(\frac{\tau -X}{\sqrt{1-R_{XY}^{2}}}\right)|D\;=0\right]\\ =\int_{-\infty }^{\infty }\Phi \left(\frac{\tau -l}{\sqrt{1-R_{{\hat{S}}_{comb}Y}^{2}}}\right)\left[\varphi \left(\frac{l-E(\gamma {\hat{S}}_{comb}|D=0)}{\sqrt{\mathrm{var}(\gamma {\hat{S}}_{comb}|D=0)}}\right)-\varphi \left(\frac{l-E(\gamma {\hat{S}}_{comb}|D=1)}{\sqrt{\mathrm{var}(\gamma {\hat{S}}_{comb}|D=1)}}\right)\right]dl\\ \;-\int_{-\infty }^{\infty }\Phi \left(\frac{\tau -l}{\sqrt{1-R_{XY}^{2}}}\right)\left[\varphi \left(\frac{l-E(X|D=0)}{\sqrt{\mathrm{var}(X|D=0)}}\right)-\varphi \left(\frac{l-E(X|D=1)}{\sqrt{\mathrm{var}(X|D=1)}}\right)\right]dl \end{array},$$

where the conditional means and variances have been obtained previously for calculation of AUC.

## 3 RESULTS

### 3.1 Cardiovascular Disease

**Figure 1:**
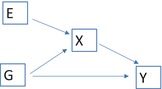
Directed acyclic graph showing correlation between polygenic and environmental scores arising from mediation *Note*: G: polygenic score; E: nongenetic risk factors; X: environmental risk score; Y: outcome.

The analytic results are now compared to some published studies of the predictive improvement from genotype data. In many countries, lipid lowering medication is advised for subjects whose medium term risk of a coronary event exceeds a threshold (Goff et al., 2014; JBS3 Board, 2014). Environmental risk scores have been developed using data from longitudinal studies, including the American College of Cardiology/American Heart Association Score (Goff et al., 2014), Framingham Risk Score (FRS) (D'Agostino et al., 2008), and QRISK‐2 equation (Hippisley‐Cox, Coupland, Robson, & Brindle, 2010). Risk factors commonly included are age, gender, components of total cholesterol, smoking, BMI, and blood pressure. A 10‐year risk of 20% has been traditionally used as a threshold although recent guidelines have suggested lower thresholds, thus widening the prescription of such medications, primarily statins (Hippisley‐Cox et al., 2010). Figure 1 is a directed acyclic graph illustrating how the environmental risk score mediates some of the genetic risk, while also including nongenetic factors.

Several studies have assessed the incremental value of a limited number of SNPs compared to traditional risk factors (Hughes et al., 2012; Morrison et al., 2007; Thanassoulis et al., 2012; Tikkanen, Havulinna, Palotie, Salomaa, & Ripatti, 2013). In general, very small improvements in AUC have been observed, but some promise has been identified for reclassification, particularly for individuals at intermediate risk. It is indeed those individuals who may be reluctant to adopt a medication and thus for whom accurate risk prediction is desirable. Two recent studies have used genome‐wide significant SNPs from the CARDIoGRAMplusC4D consortium (Deloukas et al., 2013), the largest study yet conducted in CVD, representing the state of the art in terms of the number of significantly associated SNPs and the precision of their estimated effects.

de Vries et al. (2015) considered prediction of coronary heart disease (CHD) in the Rotterdam Study using 49 SNPs significant at $P<5\times 10^{-8}$ and with 152 SNPs significant at approximately $P<10^{-3}$. Improvements over traditional risk factors were small: for example, in comparison to an environmental score including established risk factors, the AUC improved from 0.716 to 0.718 and the total NRI was 0.014 across four risk categories using the 49 SNP score. For the 152 SNP score the AUC improved to 0.719 and the NRI was 0.022.

Morris et al. (2016) considered prediction of CVD (defined as CHD or stroke) in the UCLEB consortium using the same 49 SNPs with $P<5\times 10^{-8}$ and three additional SNPs likewise associated with stroke. In comparison with the QRISK‐2 score, the AUC actually decreased from 0.635 to 0.623, whereas the NRI was 0.0025 for a 10‐year risk of 10%.

In a further recent study, Abraham et al. (2016) used all the SNPs genotyped in CARDIoGRAMplusC4D with some pruning by linkage disequilibrium. Over two target samples, AUC improved by approximately 0.016 and the continuous NRI over the FRS was between 0.147 and 0.195 in cases, and between 0.102 and 0.175 in controls.

The present model is difficult to fit to these studies because the full CARDIoGRAMplusC4D sample was genotyped on the MetaboChip, a targeted array of approximately 200,000 SNPs chosen on the basis of prior association with several cardio‐metabolic disorders. The genomic coverage and chip heritability of this array falls short of a full GWAS array; the proportion of null SNPs may be lower than in a full array, but the distribution of effect sizes will exhibit selection bias. The published list of SNP effects used by these recent studies consists of 79,138 SNPs present on both GWAS and MetaboChip products. To simplify the presentation, it is assumed that a full GWAS array was used, which could be approximated by 100,000 independent SNPs. A genetic model was identified that closely matches the results of Morris et al. (2016), which were more readily compared to the present theory than were those of the other studies. Although this model may depart from the truth, it will serve the main aim of illustrating the effects of marker selection and sample size on the incremental accuracy.

The training data from CARDIoGRAMplusC4D include 63,746 cases and 130,681 controls. Taking the chip heritability of CHD as $R_{GY}^{2}=0.3$, proportion of null SNPs $\pi_{0}=0.8$ and disease prevalence K=0.15 gives an expected AUC of 0.536 when SNPs are selected by $P<5\times 10^{-8}$, similar to the result of Morris et al. For the environmental score, the chip heritability of FRS, which includes similar risk factors to QRISK‐2, is approximately 0.3 (Simonson, Wills, Keller, & McQueen, 2011). To obtain an AUC of 0.635, as per QRISK‐2 in Morris et al., Equation (8) implies $\sigma_{X}^{2}=0.052$.

With these parameters, and selecting SNPs into the score by $P<5\times 10^{-8}$, Equation (8) gives AUC of 0.622 for the unweighted combined score when *ρ *= 0.1, or AUC of 0.621 when *ρ *= 0.4, both lower than the AUC of 0.635 for the environmental score alone and consistent with the results of Morris et al. The least squares weighted score gives AUC of 0.623 and 0.622, respectively. Such a decrease in AUC is a known effect when adding in a weakly predictive marker (Pepe, Fan, Feng, Gerds, & Hilden, 2015). However, the present theory suggests that the predictive accuracy of the unweighted score would be maximized when selecting SNPs by *P *< 0.033, giving AUC of 0.693; for the weighted score, the AUC would be 0.700 when *P *< 0.053. Clearly, in contrast to common practice, predictive accuracy is improved by more liberal selection of SNPs into the polygenic score. These results are summarized in Table 2, which also shows that under these parameters, AUC of nearly 0.8 could be achieved by the polygenic score with a very large training sample and improved only slightly by the environmental score. Supplementary Table S1 shows results with the prevalence reduced to 0.06, and Table 3 with the proportion of null SNPs increased to 0.95 (Palla & Dudbridge, 2015), both cases showing modest increase in AUC with similar qualitative conclusions.

**Table 2:** AUC for environmental score, polygenic score, and combined scores based on a genetic model matching results for CVD reported by Morris et al

	Environment	Polygenic	Unweighted Sum		Weighted Sum	
*N* cases			*ρ *= 0.1	*ρ *= 0.4	*ρ *= 0.1	*ρ *= 0.4
63,746	0.635	0.536 (5× 10^−8^)	0.622 (5× 10^−8^)	0.621 (5 × 10^−8^)	0.623 (5 × 10^−8^)	0.622 (5 × 10^−8^)
63,746	0.635	0.666 (0.053)	0.693 (0.033)	0.688 (0.036)	0.701 (0.053)	0.693 (0.053)
∞	0.635	0.782	0.800	0.784	0.800	0.788

In parentheses, *P*‐value thresholds to select SNPs into polygenic score; *N* cases, number of cases in training sample with 2.05 controls per case as in CARDIoGRAMplusC4D.

**Table 3:** NRI for a single risk threshold of 10% for combined scores based on a genetic model matching results for CVD reported by Morris et al

	Unweighted Sum				Weighted Sum			
*N* cases	*ρ *= 0.1		*ρ *= 0.4		*ρ *= 0.1		*ρ *= 0.4	
	Case	Control	Case	Control	Case	Control	Case	Control
63,746	− 0.0049	0.012	− 0.0038	0.0095	− 0.0060	0.015	0.0053	0.013
63,784	− 0.046 (0.79)	0.186 (0.031)	− 0.045 (0.76)	0.176 (0.031)	− 0.052 (0.85)	0.2 (0.056)	− 0.049 (0.86)	0.186 (0.059)
∞	− 0.046	0.367	− 0.050	0.344	− 0.046	0.368	− 0.049	0.349

In parentheses, *P*‐value thresholds to select SNPs into polygenic score; *N* cases, number of cases in training sample with 2.05 controls per case as in CARDIoGRAMplusC4D.

**Figure 2:**
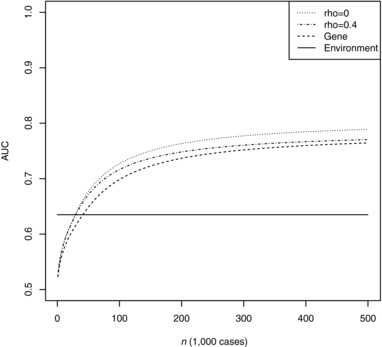
AUC of unweighted combined score as a function of training sample size *Note*: Genetic model chosen to match results for CVD reported by Morris et al., with 2.05 controls per case as in the CARDIoGRAMplusC4D consortium. Rho: chip correlation between environment and outcome. Gene: polygenic score alone. Environment: environmental score alone.

**Figure 3:**
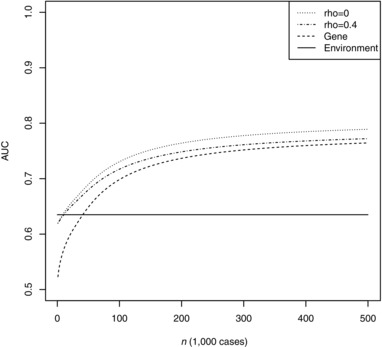
AUC of weighted combined score as a function of training sample size *Note*: Genetic model chosen to match results for CVD reported by Morris et al, with 2.05 controls per case as in the CARDIoGRAMplusC4D consortium. Rho: chip correlation between environment and outcome. Gene: polygenic score alone. Environment: environmental score alone.

Figures 2 and 3 show the expected AUC as a function of training sample size for the unweighted and least squares weighted scores. Notably, the results are almost identical for unweighted and weighted scores except at low training sample sizes. Although at a given sample size the degree of genetic correlation has small effects on AUC, it can have a strong bearing on the sample size required to reach a critical level of AUC. For example, to reach AUC of 0.75, the required sample size is approximately 159,000 when *ρ *= 0.1 but 210,000 when *ρ *= 0.4, and 284,000 for the polygenic score alone. The curves level out at around 200,000 cases, beyond which further gains are small. The CARDIoGRAMplusC4D consortium has recently exceeded this sample size and its prediction studies, when completed, will allow further refinement of these projections.

Table 3 shows expected NRI for $P<5\times 10^{-8}$ and for optimal thresholds at a single risk threshold of 10%. Morris et al. reported a case NRI of− 0.0207 and control NRI of 0.0233 for an unweighted combined score, each slightly larger in magnitude than predicted here but compatible in direction and with sampling error. Substantially larger NRI is possible with more liberal selection of SNPs, and also with a larger training sample. The improvements are largely within the controls, unaffected individuals correctly reclassified to lower risk by addition of the polygenic score. Supplementary Tables S3 and S4 show results for alternative values of the prevalence and proportion of null SNPs.

Table 4 shows results for the weighted score and *ρ *= 0.4 for the higher risk threshold of 20% and for the continuous NRI and IDI. Results (not shown) are similar for the unweighted score or *ρ *= 0.1. Again, substantial improvements are possible, though the gains now appear concentrated among cases. Supplementary Tables S5 and S6 show results for alternative values of the prevalence and proportion of null SNPs.

**Table 4:** NRI for a single‐risk threshold of 20%, continuous NRI and IDI for combined scores based on a genetic model matching results for CVD reported by Morris et al

	20% Risk		Continuous NRI		IDI
*N* cases	Case	Control	Case	Control	
63,784	0.014	− 0.006	0.080	0.014	0.0018
63,784	0.187 (0.048)	− 0.054 (0.76)	0.369 (0.053)	0.065 (0.053)	0.042 (0.053)
∞	0.347	− 0.058	0.631	0.111	0.135

In parentheses, *P*‐value thresholds to select SNPs into polygenic score; *N* cases, number of cases in training sample with 2.05 controls per case as in CARDIoGRAMplusC4D.

Note that at finite sample size, the optimal *P*‐value threshold varies according to the risk threshold and whether the case or control NRI is maximized, and those thresholds are not the ones that maximize AUC. Although the values of NRI are not greatly changed by using the threshold that maximizes AUC, this shows that the optimal genetic predictor can depend on the chosen measure of accuracy. However, the optimal threshold is the same for continuous NRI and IDI as it is for AUC.

For this model, the optimal threshold to select SNPs at the CARDIoGRAMplusC4D sample size is at approximately nominal significance. Although this is well short of genome‐wide significance, it results in fewer than 5,000 expected type‐1 errors from 100,000 tests and an expected false discovery rate of about 0.4. It is this false discovery rate, rather than the family‐wise type‐1 error, that influences the explanatory power of the polygenic score.

### 3.2 Breast cancer

Breast cancer is another disorder for which there is substantial interest in improving environmental risk scores with SNPs. Here, the general intention is to more effectively target enrolment on screening programs for early detection, rather than to directly treat risk factors. Chemoprevention may be recommended for high risk groups. A number of prediction models have been proposed, including BCRAT (commonly known at the Gail model) (Gail et al., 1989), IBIS (Tyrer‐Cuzick) (Tyrer, Duffy, & Cuzick, 2004), Barlow (Barlow et al., 2006), Rosner‐Colditz (Rosner & Colditz, 1996), and BOADICEA (Antoniou, Pharoah, Smith, & Easton, 2004). Common risk factors include age, age at menarche, mutations in *BRCA1* or *BRCA2*, age at first live birth, first degree family history, and history of breast biopsy. Generally speaking these models can perform well in women at high risk but are less accurate in the population at large.

In an early effort, Wacholder et al. (2010) estimated the improvement in the AUC of the Gail model with the addition of 10 SNPs associated from GWAS. The Gail model achieved AUC of 0.580, improving to 0.618 with the addition of SNPs. Subsequently, Darabi et al. (2012) used 18 SNPs to improve AUC from 0.548 to 0.615 in a Swedish sample. Most recently, Mavaddat et al. (2015) constructed a polygenic score from 77 SNPs identified in the largest consortium yet assembled (Michailidou et al., 2013), obtaining AUC of 0.622 with a training sample of 33,673 cases and 33,381 controls. In a simulation study, Garcia‐Closas et al. (2014) suggested this could be improved to 0.670 in combination with an environmental score with similar components to the Gail model. The environmental score alone had AUC of 0.618.

These previous studies did not consider the heritability of environmental factors such as age at menarche (Elks et al., 2010) and family history, and thus not the correlation between genetic and environmental scores. With this sample size, and again for simplicity assuming a genome‐wide panel of 100,000 independent SNPs, the 77 SNP AUC of 0.622 would be achieved when the chip heritability of breast cancer $R_{GY}^{2}=0.3$, proportion of null SNPs $\pi_{0}=0.95$ and disease prevalence K=0.05 with SNPs selected by $P<5\times 10^{-8}$. With this prevalence, an AUC of 0.618 would be achieved by an environmental score with $R_{XY}^{2}=0.0375$.

**Figure 4:**
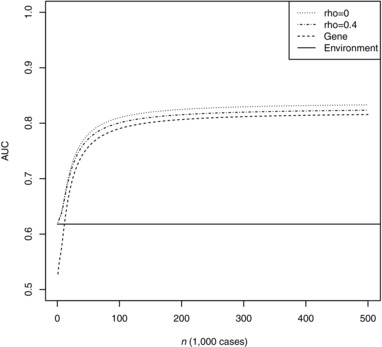
AUC of weighted combined score as a function of training sample size *Note*: Genetic model chosen to match results for breast cancer reported by Mavaddat et al., with 0.99 controls per case as in the Breast Cancer Association Consortium and $R_{GX}^{2}=0.1$. Rho: chip correlation between environment and outcome. Gene: polygenic score alone. Environment: environmental score alone.

Under these parameters, Table 5 shows expected AUC for low and high chip heritabilities of the environmental score, and low and moderate genetic correlation between disease and environment. The results for selection by $P<5\times 10^{-8}$ agree well with Garcia‐Closas et al. Again it is clear that substantial improvements are possible by more liberal selection of SNPs, and that further gains will be possible with larger training samples. Interestingly the results are quite robust to the environmental chip heritability $R_{GX}^{2}$ and the genetic correlation *ρ*. Supplementary Table S7 shows results for a higher prevalence of 0.1, and Supplementary Table S8 for a lower proportion of null SNPs of 0.8. Figure 4 shows expected AUC as a function of training sample size. Again, the gains are small even if the two scores are assumed independent, but the sample size required to reach a given AUC can depend more strongly on the genetic correlation. For example, to reach AUC of 0.8, the required sample size is about 81,000 when $R_{GX}^{2}=0.1$ and *ρ* = 0.1, but 140,000 when$R_{GX}^{2}=0.8$ and *ρ *= 0.4. The Breast Cancer Association Consortium has recently grown to about 120,000 cases, which according to Figure 4 will yield an AUC not far from the large sample limit. Again, in the near future these projections will be refined by further prediction studies.

**Table 5:** AUC for environmental score, polygenic score and combined scores based on a genetic model matching results for breast cancer reported by Mavaddat et al

	Environment	Polygenic	$R_{GX}^{2}=0.1$		$R_{GX}^{2}=0.8$	
*N* cases			ρ=0.1	ρ=0.4	ρ=0.1	ρ=0.4
33,673 (5 × 10^−8^)	0.618	0.621	0.682	0.679	0.680	0.673
33,673 (0.0035)	0.618	0.728	0.762	0.755	0.757	0.742
∞	0.618	0.820	0.840	0.832	0.835	0.821

In parentheses, *P*‐value thresholds to select SNPs into polygenic score; *N* cases, number of cases in training sample with 0.99 controls per case as in the Breast Cancer Association Consortium.

Table 6 shows NRI at a single risk threshold of 8%, which is the 10‐year absolute risk between ages 40–50 above which chemoprevention is advised in the United Kingdom (National Institute for Health and Care Excellence, 2013). Again the optimal SNP selection depends on the criterion optimized. Addition of SNPs to the Gail model would result in case NRI of 0.119 and control NRI of −0.046 when selecting SNPs by $P<5\times 10^{-8}$, improving to case NRI of 0.303 and control NRI of −0.059 with more liberal selection. The negative control NRI implies that more women would be unnecessarily recommended to receive chemoprevention, and because most women do not develop breast cancer, this translates to a large absolute number of women. Indeed the specificity for the development of cancer is 92% for the environmental score alone, but is 86% for the combined score, whereas the sensitivities are 16% and 32% respectively. Thus, while AUC and NRI appear encouraging, a large number of women would in fact be misclassified under either score. The continuous NRI shows that over half of cases are expected to increase their risk score. Supplementary Tables S9 and S10 show results for alternative values of the prevalence and proportion of null SNPs.

**Table 6:** NRI for a single‐risk threshold of 8%, continuous NRI and IDI for combined scores based on a genetic model matching results for breast cancer reported by Mavaddat et al

	8% Risk		Continuous NRI		IDI
*N* cases	Case	Control	Case	Control	
33,673 (5 × 10^−8^)	0.119	− 0.046	0.286	0.015	0.008
33,673	0.303 (0.0034)	− 0.059 (0.89)	0.544 (0.0035)	0.029 (0.0035)	0.034 (0.0035)
∞	0.441	− 0.094	0.771	0.041	0.089

In parentheses, *P*‐value thresholds to select SNPs into polygenic score; *N* cases, number of cases in training sample with 0.99 controls per case as in the Breast Cancer Association Consortium. ρ=0.4, $R_{GX}^{2}=0.8$.

Reflecting applications in screening, the breast cancer literature emphasises the proportion of cases present within some highest‐risk proportion of the population (Pharoah, Antoniou, Easton, & Ponder, 2008). This is a point on a Lorenz curve, which resembles the receiver‐operator characteristic curve with specificity replaced by a population proportion. For a proportion of the population *q* at highest risk according to score *X*, the corresponding threshold of liability is $\sqrt{\sigma_{X}^{2}}\Phi^{-1}\left(1-q\right)$ and so the proportion of cases selected by that threshold is:

$$1-\Phi \left(\frac{\sqrt{\sigma_{X}^{2}}\Phi^{-1}\left(1-q\right)-E\left(X|Y=1\right)}{\sqrt{\mathrm{var}(X|Y=1)}}\right).$$

Table 7 shows the proportion of cases within the top 10%, 20%, and 50% of the population at highest risk according to the environmental score *X* and the combined score ${\hat{S}}_{comb}$. These results suggest, for example, that at current sample sizes nearly half of cases could be detected by screening the 20% of the population with highest combined scores. With larger training samples, over 90% of cases might be detected by screening the half of the population with highest scores. These results are compatible with those of previous studies (Garcia‐Closas et al., 2014; Pharoah et al., 2008). Supplementary Tables S11 and S12 show results for alternative values of the prevalence and proportion of null SNPs. Similar to other sensitivity analyses in the supplementary tables, these yield modest quantitative changes with similar qualitative conclusions.

**Table 7:** Proportion of cases present among highest risk quantiles in the population under a genetic model matching results reported in Mavaddat et al

	Top 10%			Top 20%			Top 50%		
*N* cases	Polygenic	Env	Comb	Polygenic	Env	Comb	Polygenic	Env	Comb
33,673 (5 × 10^−8^)	18.8	18.5	22.2	33.0	32.7	37.7	66.2	65.7	71.1
33,673 (0.0035)	29.8	18.5	30.7	47.6	32.7	48.7	79.9	65.7	80.9
∞	43.0	18.5	43.0	63.1	32.7	63.1	90.5	65.7	90.5

Polygenic: polygenic score alone; Env: environmental score alone; Comb: least squares weighted sum; in parentheses: *P*‐value thresholds to select SNPs into polygenic score; *N* cases: number of cases in training sample with 0.99 controls per case as in the Breast Cancer Association Consortium.

### 3.3 Height

Here the example of height is used to illustrate a relationship between a polygenic score and family history. Of course, family history is an environmental risk factor for any heritable condition, but it will be correlated with the measured genetic risk. An initial challenge for any genetic predictor is to exceed the predictive accuracy of family history (Do, Hinds, Francke, & Eriksson, 2012). For example, Aulchenko et al. (2009) showed that 54 associated SNPs could not predict an individual's height as accurately as could the mean height of its parents. Through simulation they showed that a gene score explaining all the heritability could predict better than the family history. A natural question is: what sample size would allow development of a gene score with predictive accuracy better than the family history?

**Figure 5:**
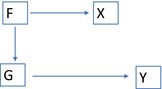
Directed acyclic graph showing correlation between polygenic and environmental scores arising from family history A simplified scenario is shown in which there are no shared environmental effects. G: polygenic score; F: polygenic scores of other family members; X: family history; Y: outcome.

Figure 5 is a directed acyclic graph showing a simplified situation in which the family history is entirely explained by genetics. Although in many cases shared environment also contributes to family history, this graph may be fairly appropriate for height because it is highly heritable and parents have often reached their full stature before producing offspring. Under this structure, the parameters of the quantitative model may be derived exactly in terms of the heritability.

Assume the commonly used value of 80% for the heritability of height. Under an additive model, the height of a child is the sum of genetic contributions from mother and father and of unique environmental contributions. It is easy to show that the genetic covariance between the child and the mean‐parents is half the heritability, and then that the genetic correlation, $\rho =\frac{1}{\sqrt{2}}$. Furthermore the variance in child explained by the mean‐parents is twice the square of the covariance, so $R_{XY}^{2}=0.32$, which is taken as the predictive accuracy of the family history. Finally, the chip heritability of the mean‐parents is half the chip heritability, $R_{GX}^{2}=\frac{1}{2}R_{GY}^{2}$.

The GIANT consortium has conducted discovery meta‐analyses for height, which despite very large samples have yet to exceed the predictive accuracy of family history. A discovery sample of approximately 134,000 achieved *R* ^2^ of 13.3% by selecting SNPs with *P *< 5 × 10^−4^ (Lango Allen et al., 2010); a larger study of approximately 250,000 improved only to *R* ^2^ of 17% with *P *< 5 × 10^−5^ (Wood et al., 2014). These studies used a densely imputed panel of 2.5 M SNPs with chip heritability estimated within contributing studies at 60% of the total heritability, so $R_{GY}^{2}=0.6\times 0.8=0.48$. Stepwise regressions were used to estimate SNP weights, so the estimated effects may be considered approximately independent across all 2.5 M SNPs. Across six polygenic scores with different selection thresholds, the best fitting model (Palla & Dudbridge, 2015) has chip heritability $R_{GY}^{2}=0.34$ and proportion of null SNPs $\pi_{0}=0.996$. This consortium level estimate of $R_{GY}^{2}$ is lower than the study‐level estimates of 0.48, as has previously been observed (Yang et al., 2015). This is likely due to heterogeneity in data management across studies, if not actual genetic heterogeneity, and can be accounted for by estimating the genetic covariance between training and target data as a free parameter separately from the chip heritability in the training data (Palla & Dudbridge, 2015). This yields a genetic covariance of 0.46 with $\pi_{0}=0.995$; the estimate of $R_{GY}^{2}$ is 1, but this is a known artifact of this method, which has little bearing on estimates of predictive accuracy. Under this model, the prediction *R* ^2^ is 0.21 with an infinite training sample, implying that the consortium‐based estimate can never predict as well as the family history.

This alarming result is mitigated by considering a homogeneous training sample in which we assume $R_{GY}^{2}=0.48$, with $\pi_{0}=0.995$ as estimated above and perfect genetic correlation with the target sample. Then *R* ^2 ^> 0.32 when the training sample exceeds 301,000 subjects. Emerging datasets such as UK Biobank will soon allow such predictions to be tested empirically.

**Figure 6:**
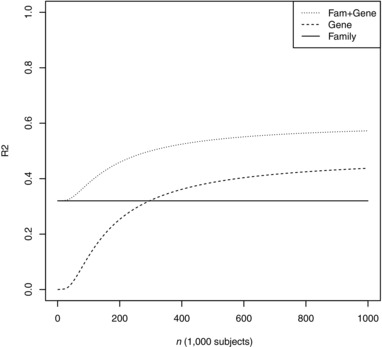
Prediction *R* ^2^ as a function of training sample size *Note*: Genetic model chosen to match results for height reported by the GIANT consortium. Family: mean‐parental height. Gene: polygenic score alone. Fam + Gene: least squares weighted combination of polygenic score and mean‐parental height

Under this model, Figure 6 shows the accuracy of a combined score as a function of training sample size, showing that the family history continues to provide useful information even as the polygenic risk becomes more informative. This is not surprising, as the chip heritability falls short of the total heritability, which can be accessed, albeit imperfectly, by the family history. Nevertheless, this vignette demonstrates that an independent effect of family history is consistent with it being entirely genetic in origin, as long as the genetic predictor is not complete. Furthermore, it is apparent that for strongly heritable traits, the high threshold of predictive accuracy set by family history can only be overcome through very large and homogeneous genetic discovery studies.

## 4 DISCUSSION

Accurate genetic prediction of disease risk remains an elusive goal, but its importance is evident from an increasing number of studies evaluating its potential for many traits. In conjunction with existing environmental predictors, the incremental benefit of polygenic scores has appeared modest to date. The results presented here allow an informed interpretation of observed results and anticipation of future results as larger training datasets are assembled. In particular, it is clear that at current sample sizes predictive accuracy, and incremental benefit, could be substantially improved by including SNPs at liberal significance thresholds. Furthermore, for chip correlation up to 0.4, the degree of correlation and the heritability of the environmental score have little bearing on predictive accuracy. These results are encouraging for practice because they reduce concern about accounting for the heritable components of environmental scores. In particular, it seems acceptable to combine consortium estimates of marginal SNP effects with environmental risk scores, rather than jointly estimating conditional SNP and environmental effects in training samples that may be much smaller than those of consortia.

The majority of studies to date have restricted their polygenic scores to genome‐wide significant SNPs, but this is suboptimal. One justification has been that genotyping a limited number of SNPs is more cost‐effective than a whole‐genome panel; but this should be viewed in the context of potentially predicting multiple conditions from a single DNA sample, for which genome‐wide typing will be more efficient. Another view is that it is difficult to argue for inclusion of individual risk factors (here SNPs) that have but weak evidence of association. However, by viewing the polygenic risk as a single entity, this argument becomes irrelevant; the role of individual SNPs then applies only to how that risk is calculated.

The present work unifies previous work of So and Sham (2010), who showed how many common measures of predictive accuracy can be expressed in terms of liability *R* ^2^, with that of Dudbridge (2013), who allowed for finite training samples and selection of SNPs into the polygenic score. Only the most commonly reported measures are considered here, namely AUC, NRI, and IDI, but other measures, such as those related to predictiveness curves (Pepe et al., 2008) and net benefit (Baker, 2009), could be treated in the same manner. In contrast to some previous approaches, the genetic covariance between environmental score and predicted trait is considered here explicitly, along with unweighted and weighted combinations of genetic and environmental scores. Although the genetic covariance is the key property, the results are presented in terms of genetic correlation and variances, as these quantities can be estimated readily by existing methods (Bulik‐Sullivan et al., 2015; Lee et al., 2012).

The modest effect of genetic correlation on AUC is not too surprising, because AUC depends on ranking the scores rather than their absolute values, but more surprisingly the NRI, which does depend on absolute risk, is also quite robust to correlation. Higher levels of correlation will of course tend toward no incremental benefit, but such scenarios are arguably unlikely because most environmental scores include nonheritable factors such as age, as well as the environmental component of the heritable factors. Nevertheless, the predictive accuracy can be improved by accounting for covariance between genetic and environmental predictors. Furthermore, the level of correlation can have more substantial effects on the sample size required to reach a prespecified level of accuracy.

The NRI has been criticized on theoretical and practical grounds (Kerr et al., 2014; Pepe et al., 2015), yet it has an intuitive appeal that should not be overlooked. An important recommendation is that it should be reported separately for cases and controls, especially if they are associated with different costs (Pencina et al., 2011). Here, it has been shown that for given training data, the optimal polygenic score differs for case NRI and control NRI, and also varies with risk thresholds. Because in a prospective setting one cannot know who is a case, the polygenic score must be defined according to a criterion based on relative costs of case and control NRI. Such considerations will further complicate reporting of NRI in evaluation studies, although this problem does not apply to the continuous NRI or IDI.

We have focused on two commonly reported measures, AUC and NRI, yet both measures have been severely criticized and there is a recognized need for more appropriate measures of incremental benefit. For example, stratification of the population by genetic risk may allow more efficient application of environmental risk scores. In CVD, a group at high genetic risk attained 20% estimated risk at an age up to 18 years younger than the low genetic risk group (Abraham et al., 2016). In breast cancer, the absolute risk associated with modifiable risk factors was significantly higher among women in the top decile of nonmodifiable risk (including 92 SNPs) compared to those in the lowest decile (Maas et al., 2016). These results suggest that even limited genetic data can provide useful stratification for identifying subjects who would benefit most from intervention. Such perspectives offer a more optimistic view of genetic prediction that will only become stronger with larger training samples and more liberal selection of genetic markers.

Consortium studies are now approaching the sizes at which useful levels of predictive accuracy are predicted by theory. However, such results have not yet been achieved in practice. One clear reason is that many studies have restricted their polygenic scores to genome‐wide significant SNPs. Given the large size of the CARIODoGRAMplusC4D consortium, higher levels of accuracy should be possible by including more SNPs in the risk score. This has been demonstrated by Abraham et al. (2016), although their approach of pruning the full set of SNPs may be suboptimal. Furthermore, accurate odds ratios are currently available only for a targeted array product, the MetaboChip, further limiting the accuracy that can be achieved. Therefore their results, which are intermediate between those using genome‐wide significant SNPs and those predicted here, seem consistent with the present theory. The present work suggests that as genome‐wide typing is completed on a larger number of studies, more accurate prediction will be achieved by highly polygenic scores.

The Breast Cancer Association Consortium too is approaching a sufficiently large number of cases. To date, consortium‐wide genotypes have only been generated on the iCOGS platform, another targeted array focused on candidate genes for certain cancers. Again therefore, prediction studies have focused on genome‐wide significant SNPs, but the next generation of targeted arrays, which include a genome‐wide backbone, offer greater promise for developing more accurate polygenic scores.

Given the large size of the GIANT consortium, the continuing modest prediction of height is surprising but could be explained by heterogeneity between consortium‐level training data and individual target studies. The source of this heterogeneity remains unclear (Yang et al., 2015), but with the emergence of large, homogeneous datasets such as UK Biobank, more accurate genetic predictors could be developed that exceed the predictive accuracy of the family history.

An alternative explanation is that the present model for genetic effects, consisting of a mixture of a normal distribution and a mass at zero, is incorrect for this phenotype. If in fact the true distribution had a sharp peak at zero, then the present model could fit the data well, but the individual SNP effects would be more dispersed and thus harder to estimate *en masse*. Such a model could apply, for example, to schizophrenia, for which fine‐scale heritability analyses suggest an extremely polygenic architecture (Loh et al., 2015), while the best fitting normal‐null mixture estimates a null proportion of around 90% (Palla & Dudbridge, 2015). Although theory based on a mixture of exponential distributions (Chatterjee et al., 2013) has roughly agreed with the normal‐null mixture, there is a notable exception in the case of height, for which a mixture of three exponentials fits GIANT data well with chip heritability at 45%. This fitted model projects that prediction *R* ^2^ will not exceed 0.32 until the training sample size exceeds one million, in contrast to our projection of 301,000 for a homogeneous sample. Finally, departures from the additive model may lead to a poor fit of the polygenic score.

The substantial heritability of most common disorders implies that clinically useful prediction can be achieved when the heritable risk is accurately measured. The present results suggest that such results may not be too far off in terms of discovery sample size. As that level is approached, the polygenic score dominates the prediction compared to existing environmental scores, although the dynamic and potentially modifiable nature of environmental factors ensures that they will continue to play a crucial role in assessing absolute risk. Such predictors may include epigenetic or other “omic” factors that themselves are selected from high dimensional panels. The present work assumes a known, low dimensional risk factor whose effect on outcome is known precisely. Extension to high dimensional, dynamic nongenetic predictors, allowing for simple model selection as in the polygenic score, will be pursued in subsequent work.

## Supporting information

Table S1. AUC for environmental score, polygenic score and combined scores. Genetic model is identical to that in main Table 2 except that the prevalence is reduced from 0.15 to 0.06. In parentheses, *P*‐value thresholds to select SNPs into polygenic score. N cases, number of cases in training sample with 2.05 controls per case as in CARDIoGRAMplusC4D.
Table S2. AUC for environmental score, polygenic score and combined scores. Genetic model is identical to that in main Table 2 except that the proportion of null SNPs is increased from 0.8 to 0.95. In parentheses, *P*‐value thresholds to select SNPs into polygenic score. N cases, number of cases in training sample with 2.05 controls per case as in CARDIoGRAMplusC4D.
Table S3. NRI for a single risk threshold of 10% for combined. Genetic model is identical to that in main Table 3 except that the prevalence is reduced from 0.15 to 0.06. In parentheses, *P*‐value thresholds to select SNPs into polygenic score. N cases, number of cases in training sample with 2.05 controls per case as in CARDIoGRAMplusC4D.
Table S4. NRI for a single risk threshold of 10% for combined. Genetic model is identical to that in main Table 3 except that the proportion of null SNPs is increased from 0.8 to 0.95. In parentheses, *P*‐value thresholds to select SNPs into polygenic score. N cases, number of cases in training sample with 2.05 controls per case as in CARDIoGRAMplusC4D.
Table S5. NRI for a single risk threshold of 20%, continuous NRI and IDI for combined scores. Genetic model is identical to that in main Table 4 except that the prevalence is reduced from 0.15 to 0.06. In parentheses, *P*‐value thresholds to select SNPs into polygenic score. N cases, number of cases in training sample with 2.05 controls per case as in CARDIoGRAMplusC4D.
Table S6. NRI for a single risk threshold of 20%, continuous NRI and IDI for combined scores. Genetic model is identical to that in main Table 4 except that the proportion of null SNPs is increased from 0.8 to 0.95. In parentheses, *P*‐value thresholds to select SNPs into polygenic score. N cases, number of cases in training sample with 2.05 controls per case as in CARDIoGRAMplusC4D.
Table S7. AUC for environmental score, polygenic score and combined scores. Genetic model is identical to that in main Table 5 except that the prevalence is increased from 0.05 to 0.1. In parentheses, *P*‐value thresholds to select SNPs into polygenic score. N cases, number of cases in training sample with 0.99 controls per case as in the Breast Cancer Association Consortium.
Table S8. AUC for environmental score, polygenic score and combined scores. Genetic model is identical to that in main Table 5 except that the proportion of null SNPs is decreased from 0.95 to 0.8. In parentheses, *P*‐value thresholds to select SNPs into polygenic score. N cases, number of cases in training sample with 0.99 controls per case as in the Breast Cancer Association Consortium.
Table S9. NRI for a single risk threshold of 8%, continuous NRI and IDI for combined scores. Genetic model is identical to that in main Table 6 except that the prevalence is increased from 0.05 to 0.1. In parentheses, *P*‐value thresholds to select SNPs into polygenic score. N cases, number of cases in training sample with 0.99 controls per case as in the Breast Cancer Association Consortium.
Table S10. NRI for a single risk threshold of 8%, continuous NRI and IDI for combined scores. Genetic model is identical to that in main Table 5 except that the proportion of null SNPs is decreased from 0.95 to 0.8. In parentheses, *P*‐value thresholds to select SNPs into polygenic score. N cases, number of cases in training sample with 0.99 controls per case as in the Breast Cancer Association Consortium.
Table S11. Proportion of cases present among highest risk quantiles in the population. Genetic model is identical to that in main Table 6 except that the prevalence is increased from 0.05 to 0.1. Polyg, polygenic score alone. Env, environmental score alone. Comb, least squares weighted sum. In parentheses, *P*‐value thresholds to select SNPs into polygenic score. N cases, number of cases in training sample with 0.99 controls per case as in the Breast Cancer Association Consortium.
Table S12. Proportion of cases present among highest risk quantiles in the population. Genetic model is identical to that in main Table 5 except that the proportion of null SNPs is decreased from 0.95 to 0.8. Polyg, polygenic score alone. Env, environmental score alone. Comb, least squares weighted sum. In parentheses, *P*‐value thresholds to select SNPs into polygenic score. N cases, number of cases in training sample with 0.99 controls per case as in the Breast Cancer Association Consortium.
