# Supplementary material for: Predictive accuracy of combined genetic and environmental risk scores
Source: Genet Epidemiol. 2017 Nov 26;42(1):4–19. doi: 10.1002/gepi.22092 (PMC5847122; doi:10.1002/gepi.22092)
Supplement: Supplementary file 1 — Table S1. AUC for environmental score, polygenic score and combined scores. Genetic model is identical to that in main Table 2 except that the prevalence is reduced from 0.15 to 0.06. In parentheses, P‐value thresholds to select SNPs into polygenic score. N cases, number of cases in training sample with 2.05 controls per case as in CARDIoGRAMplusC4D. Table S2. AUC for environmental score, polygenic score and combined scores. Genetic model is identical to that in main Table 2 except that the proportion of null SNPs is increased from 0.8 to 0.95. In parentheses, P‐value thresholds to select SNPs into polygenic score. N cases, number of cases in training sample with 2.05 controls per case as in CARDIoGRAMplusC4D. Table S3. NRI for a single risk threshold of 10% for combined. Genetic model is identical to that in main Table 3 except that the prevalence is reduced from 0.15 to 0.06. In parentheses, P‐value thresholds to select SNPs into polygenic score. N cases, number of cases in training sample with 2.05 controls per case as in CARDIoGRAMplusC4D. Table S4. NRI for a single risk threshold of 10% for combined. Genetic model is identical to that in main Table 3 except that the proportion of null SNPs is increased from 0.8 to 0.95. In parentheses, P‐value thresholds to select SNPs into polygenic score. N cases, number of cases in training sample with 2.05 controls per case as in CARDIoGRAMplusC4D. Table S5. NRI for a single risk threshold of 20%, continuous NRI and IDI for combined scores. Genetic model is identical to that in main Table 4 except that the prevalence is reduced from 0.15 to 0.06. In parentheses, P‐value thresholds to select SNPs into polygenic score. N cases, number of cases in training sample with 2.05 controls per case as in CARDIoGRAMplusC4D. Table S6. NRI for a single risk threshold of 20%, continuous NRI and IDI for combined scores. Genetic model is identical to that in main Table 4 except that the proportion of null SNPs is increased from 0.8 to 0.95. [file GEPI-42-4-s001.docx]

# Predictive accuracy of combined genetic and environmental risk scores

Frank Dudbridge, Nora Pashayan, Jian Yang

## Supplementary tables

Table S1. AUC for environmental score, polygenic score and combined scores. Genetic model is identical to that in main Table 2 except that the prevalence is reduced from 0.15 to 0.06. In parentheses, *P*-value thresholds to select SNPs into polygenic score. N cases, number of cases in training sample with 2.05 controls per case as in CARDIoGRAMplusC4D.

|  | Environment | Polygenic | Unweighted sum | | Weighted sum | |
| --- | --- | --- | --- | --- | --- | --- |
| N cases |  |  | *ρ*=0.1 | *ρ*=0.4 | *ρ*=0.1 | *ρ*=0.4 |
| 63746 | 0.635 | 0.565 (5e-8) | 0.647 (5e-8) | 0.645 (5e-8) | 0.649 (5e-8) | 0.647 (5e-8) |
| 63746 | 0.635 | 0.713 (.039) | 0.742 (.029) | 0.734 (.033) | 0.746 (.039) | 0.736 (.039) |
| ∞ | 0.635 | 0.814 | 0.832 | 0.816 | 0.832 | 0.820 |

Table S2. AUC for environmental score, polygenic score and combined scores. Genetic model is identical to that in main Table 2 except that the proportion of null SNPs is increased from 0.8 to 0.95. In parentheses, *P*-value thresholds to select SNPs into polygenic score. N cases, number of cases in training sample with 2.05 controls per case as in CARDIoGRAMplusC4D.

|  | Environment | Polygenic | Unweighted sum | | Weighted sum | |
| --- | --- | --- | --- | --- | --- | --- |
| N cases |  |  | *ρ*=0.1 | *ρ*=0.4 | *ρ*=0.1 | *ρ*=0.4 |
| 63746 | 0.635 | 0.664 (5e-8) | 0.699 (5e-8) | 0.691 (5e-8) | 0.699 (5e-8) | 0.691 (5e-8) |
| 63746 | 0.635 | 0.733 (.003) | 0.757 (.003) | 0.746 (.003) | 0.757 (.003) | 0.746 (.003) |
| ∞ | 0.635 | 0.782 | 0.800 | 0.784 | 0.800 | 0.788 |

Table S3. NRI for a single risk threshold of 10% for combined. Genetic model is identical to that in main Table 3 except that the prevalence is reduced from 0.15 to 0.06. In parentheses, *P*-value thresholds to select SNPs into polygenic score. N cases, number of cases in training sample with 2.05 controls per case as in CARDIoGRAMplusC4D.

|  | Unweighted sum | | | | Weighted sum | | | | |
| --- | --- | --- | --- | --- | --- | --- | --- | --- | --- |
| N cases | *ρ*=0.1 | | *ρ*=0.4 | | *ρ*=0.1 | | *ρ*=0.4 | | |
|  | Case | Control | Case | Control | Case | Control | | Case | Control |
| 63746 | 0.036 | -0.014 | 0.03 | -0.012 | 0.039 | -0.015 | | 0.035 | -0.013 |
| 63784 | 0.269 (.034) | -0.065 (.76) | 0.253 (.034) | -0.063 (.77) | 0.278 (.04) | -0.07 (.76) | | 0.257 (.038) | -0.067 (.76) |
| ∞ | 0.446 | -0.078 | 0.417 | -0.080 | 0.446 | -0.078 | | 0.424 | -0.080 |

Table S4. NRI for a single risk threshold of 10% for combined. Genetic model is identical to that in main Table 3 except that the proportion of null SNPs is increased from 0.8 to 0.95. In parentheses, *P*-value thresholds to select SNPs into polygenic score. N cases, number of cases in training sample with 2.05 controls per case as in CARDIoGRAMplusC4D.

|  | Unweighted sum | | | | Weighted sum | | | | |
| --- | --- | --- | --- | --- | --- | --- | --- | --- | --- |
| N cases | *ρ*=0.1 | | *ρ*=0.4 | | *ρ*=0.1 | | *ρ*=0.4 | | |
|  | Case | Control | Case | Control | Case | Control | | Case | Control |
| 63746 | -0.053 | 0.02 | -0.051 | 0.181 | -0.053 | 0.197 | | -0.051 | 0.181 |
| 63784 | -0.046 (.92) | 0.3 (.003) | -0.044 (.96) | 0.281 (.002) | -0.051 (.78) | 0.3 (.003) | | -0.049 (.95) | 0.281 (.003) |
| ∞ | -0.046 | 0.367 | -0.050 | 0.344 | -0.046 | 0.368 | | -0.049 | 0.349 |

Table S5. NRI for a single risk threshold of 20%, continuous NRI and IDI for combined scores. Genetic model is identical to that in main Table 4 except that the prevalence is reduced from 0.15 to 0.06. In parentheses, *P*-value thresholds to select SNPs into polygenic score. N cases, number of cases in training sample with 2.05 controls per case as in CARDIoGRAMplusC4D.

|  | 20% risk | | Continuous NRI | | IDI |
| --- | --- | --- | --- | --- | --- |
| N cases | Case | Control | Case | Control |  |
| 63784 | 0.003 | -0.0007 | 0.156 | 0.01 | 0.0028 |
| 63784 | 0.107 (.04) | -0.016 (.85) | 0.513 (.039) | 0.033 (.039) | 0.036 (.039) |
| ∞ | 0.307 | -0.046 | 0.750 | 0.048 | 0.097 |

Table S6. NRI for a single risk threshold of 20%, continuous NRI and IDI for combined scores. Genetic model is identical to that in main Table 4 except that the proportion of null SNPs is increased from 0.8 to 0.95. In parentheses, *P*-value thresholds to select SNPs into polygenic score. N cases, number of cases in training sample with 2.05 controls per case as in CARDIoGRAMplusC4D.

|  | 20% risk | | Continuous NRI | | IDI |
| --- | --- | --- | --- | --- | --- |
| N cases | Case | Control | Case | Control |  |
| 63784 | 0.181 | -0.056 | 0.361 | 0.064 | 0.04 |
| 63784 | 0.28 (.004) | -0.054 (.82) | 0.517 (.003) | 0.091 (.003) | 0.086 (.003) |
| ∞ | 0.347 | -0.058 | 0.631 | 0.111 | 0.135 |

Table S7. AUC for environmental score, polygenic score and combined scores. Genetic model is identical to that in main Table 5 except that the prevalence is increased from 0.05 to 0.1. In parentheses, *P*-value thresholds to select SNPs into polygenic score. N cases, number of cases in training sample with 0.99 controls per case as in the Breast Cancer Association Consortium.

|  | Environment | Polygenic | $R_{GX}^{2}=0.1$ |  | $R_{GX}^{2}=0.8$ |  |
| --- | --- | --- | --- | --- | --- | --- |
| N cases |  |  | $\rho=0.1$ | $\rho=0.4$ | $\rho=0.1$ | $\rho=0.4$ |
| 33,673 (5e-8) | 0.618 | 0.586 | 0.636 | 0.634 | 0.635 | 0.631 |
| 33,673 (0.0037) | 0.618 | 0.690 | 0.715 | 0.710 | 0.712 | 0.700 |
| ∞ | 0.618 | 0.796 | 0.810 | 0.803 | 0.806 | 0.796 |

Table S8. AUC for environmental score, polygenic score and combined scores. Genetic model is identical to that in main Table 5 except that the proportion of null SNPs is decreased from 0.95 to 0.8. In parentheses, *P*-value thresholds to select SNPs into polygenic score. N cases, number of cases in training sample with 0.99 controls per case as in the Breast Cancer Association Consortium.

|  | Environment | Polygenic | $R_{GX}^{2}=0.1$ |  | $R_{GX}^{2}=0.8$ |  |
| --- | --- | --- | --- | --- | --- | --- |
| N cases |  |  | $\rho=0.1$ | $\rho=0.4$ | $\rho=0.1$ | $\rho=0.4$ |
| 33,673 (5e-8) | 0.618 | 0.515 | 0.619 | 0.619 | 0.619 | 0.619 |
| 33,673 (0.192) | 0.618 | 0.647 | 0.686 | 0.683 | 0.684 | 0.675 |
| ∞ | 0.618 | 0.820 | 0.834 | 0.826 | 0.830 | 0.820 |

Table S9. NRI for a single risk threshold of 8%, continuous NRI and IDI for combined scores. Genetic model is identical to that in main Table 6 except that the prevalence is increased from 0.05 to 0.1. In parentheses, *P*-value thresholds to select SNPs into polygenic score. N cases, number of cases in training sample with 0.99 controls per case as in the Breast Cancer Association Consortium.

|  | 8% risk | | Continuous NRI | | IDI |
| --- | --- | --- | --- | --- | --- |
| N cases | Case | Control | Case | Control |  |
| 33,673 (5e-8) | -0.019 | 0.059 | 0.193 | 0.021 | 0.007 |
| 33,673 | -0.027 (.910) | 0.179 (.0036) | 0.431 (.0037) | 0.048 (.0037) | 0.038 (.0037) |
| ∞ | 0.009 | 0.299 | 0.691 | 0.077 | 0.116 |

Table S10. NRI for a single risk threshold of 8%, continuous NRI and IDI for combined scores. Genetic model is identical to that in main Table 5 except that the proportion of null SNPs is decreased from 0.95 to 0.8. In parentheses, *P*-value thresholds to select SNPs into polygenic score. N cases, number of cases in training sample with 0.99 controls per case as in the Breast Cancer Association Consortium.

|  | 8% risk | | Continuous NRI | | IDI |
| --- | --- | --- | --- | --- | --- |
| N cases | Case | Control | Case | Control |  |
| 33,673 (5e-8) | 0.002 | -0.001 | 0.034 | 0.002 | 0.00007 |
| 33,673 | 0.163 (.218) | -0.059 (.735) | 0.348 (.192) | 0.018 (.192) | 0.014 (.434) |
| ∞ | 0.463 | -0.093 | 0.771 | 0.041 | 0.089 |

Table S11. Proportion of cases present among highest risk quantiles in the population. Genetic model is identical to that in main Table 6 except that the prevalence is increased from 0.05 to 0.1. Polyg, polygenic score alone. Env, environmental score alone. Comb, least squares weighted sum. In parentheses, *P*-value thresholds to select SNPs into polygenic score. N cases, number of cases in training sample with 0.99 controls per case as in the Breast Cancer Association Consortium.

|  | Top 10% | | | Top 20% | | | Top 50% | | |
| --- | --- | --- | --- | --- | --- | --- | --- | --- | --- |
| N cases | Polyg | Env | Comb | Polyg | Env | Comb | Polyg | Env | Comb |
| 33,673 (5e-8) | 15.5 | 18.5 | 18.8 | 28.4 | 32.7 | 33.2 | 61.0 | 65.7 | 66.6 |
| 33,673 (0.0037) | 23.9 | 18.5 | 24.9 | 40.3 | 32.7 | 41.7 | 73.9 | 65.7 | 75.2 |
| ∞ | 35.6 | 18.5 | 35.6 | 55.5 | 32.7 | 55.5 | 86.6 | 65.7 | 86.6 |

Table S12. Proportion of cases present among highest risk quantiles in the population. Genetic model is identical to that in main Table 5 except that the proportion of null SNPs is decreased from 0.95 to 0.8. Polyg, polygenic score alone. Env, environmental score alone. Comb, least squares weighted sum. In parentheses, *P*-value thresholds to select SNPs into polygenic score. N cases, number of cases in training sample with 0.99 controls per case as in the Breast Cancer Association Consortium.

|  | Top 10% | | | Top 20% | | | Top 50% | | |
| --- | --- | --- | --- | --- | --- | --- | --- | --- | --- |
| N cases | Polyg | Env | Comb | Polyg | Env | Comb | Polyg | Env | Comb |
| 33,673 (5e-8) | 10.9 | 18.5 | 18.6 | 21.4 | 32.7 | 32.7 | 52.0 | 65.7 | 65.9 |
| 33,673 (0.192) | 21.1 | 18.5 | 23.8 | 40.3 | 36.3 | 39.9 | 69.6 | 65.7 | 73.2 |
| ∞ | 43.0 | 18.5 | 43.0 | 63.1 | 32.7 | 63.1 | 90.5 | 65.7 | 90.5 |
